# The Great Resignation's Impact on Nursing: A Phenomenological Study

**DOI:** 10.1111/inr.70046

**Published:** 2025-06-13

**Authors:** Jolene Kittle

**Affiliations:** ^1^ Decker College of Nursing and Health Sciences Binghamton University Binghamton New York USA

**Keywords:** great resignation, nursing, nursing leadership, practice, retention

## Abstract

**Aim:**

The aim of this study is to explore the lived experiences of nurses practicing during and after the phenomenon known as the “Great Resignation” through the lens of the Motivation to Work theory.

**Background/Introduction:**

The number of workers quitting their jobs since the COVID‐19 pandemic reached record highs and this time period is often referred to as the “Great Resignation.” There is a need to understand this phenomenon in nursing.

**Methods:**

This study used a transcendental phenomenological design and adhered to the Consolidated Criteria for Reporting Qualitative Research guidelines.

**Findings:**

The experiences of 18 registered nurses practicing during the “Great Resignation” were summarized in five themes: why I am a nurse, work environment, human needs, autonomy, and COVID.

**Discussion:**

This study highlights that nurses practicing during the “Great Resignation” have stayed in the profession because of their commitment to their patients. However, increasing workload demands leave them feeling like they no longer can deliver the care their patients deserve. Nurses reported feeling that better resources to support the practice of nursing are needed and that how healthcare is delivered needs to be redesigned.

**Conclusion:**

Nurses chose the profession to help people and deliver high quality care. Work environment factors are creating barriers to deliver this care and nurses feel a lack of leadership support, autonomy, appreciation, and value.

**Implications for Nursing and Health Policy:**

Findings from this study can inform policy surrounding a healthy work environment, retention strategies, and sustainability of the nursing profession.

## 1 Introduction

The number of workers quitting their jobs in the years since the COVID‐19 pandemic reached rates so high that economists often refer to this time period as the “The Great Resignation.” Coined by Anthony Klotz (Thompson 2021), this phenomenon is defined by employees resigning from their jobs in mass numbers between the years 2021 and 2023 (Melhorn and Hoover 2024). In the general workforce, workers were leaving for entrepreneurial ventures and freelancing in search of better work–life balance, improved wages, and opportunities for career growth (Shukla et al. 2022). During this time, nurses were also leaving their jobs at a high rate (Boston‐Fleischhauer 2022; Hagans 2021; Johnson 2022; Poindexter 2022). Globally, 20%–38% of nurses in the United Kingdom, Singapore, Japan, France, and Australia reported intention to leave their role in direct care in the next year (Berlin et al. 2022). In the United States, 32% of nurses reported a plan to leave their direct care position (Berlin et al. 2022). In the report published by the National Council of State Boards of Nursing (NCSBN) titled, *NCSBN Research Projects Significant Nursing Workforce Shortages and Crisis* (2023), 100,000 registered nurses (RNs) resigned in the two years after the pandemic and 610,388 are expected to leave by 2027. Studies conducted during this time reported that nurses were experiencing an increase in workload (Smiley et al. 2023), feeling burned out, fatigued, “used up,” and emotionally drained (Martin et al. 2023). No research exists focused on the experiences of RNs practicing during the “Great Resignation” and their motivations to remain in the profession of nursing.

The Motivation to Work theory (Herzberg et al. 1993) was the framework that guided this study. This theory posits factors including salary, working conditions, and policies—many of the reasons cited by nurses for leaving must be addressed to reduce dissatisfaction. Reducing dissatisfaction alone does not increase workplace satisfaction, which has been shown to be one of the strongest motivations to stay (Lee and Lee 2022). To increase satisfaction, factors such as growth and advancement potential, achievement, recognition, and responsibility must be addressed.

### 1.1 Aim of the study

The aim of this study was to explore the lived experience of RNs practicing during the “Great Resignation” and their motivations to stay through the lens of the Motivation to Work theory (Herzberg et al. 1993).

## 2 Methods

### 2.1 Research Design

A transcendental phenomenological design was utilized in this study (Moustakas 2009). This design was chosen because of the focus on understanding the human experience of a phenomenon, adding depth by examining the conditions that make that experience possible. The profession of nursing is a human experience. Phenomenological studies in nursing promote a comprehensive understanding of the lived experience of humans beyond the numbers (Norlyk et al. 2023). Examples of previous phenomenological studies in nursing have focused on fathers caring for neonates (Hemle Jerntorp et al. 2021), women's needs with breast cancer (Arikan Dönmez et al. 2021), mental health, and aging (Al Gilani et al. 2021). This study adhered to the Consolidated Criteria for Reporting Qualitative Research guidelines.

### 2.2 Sample and Setting

Participants were recruited via an email announcement on the listserv to one international nursing organization (Sigma Theta Tau International) and one social media listserv (LinkedIn). Potential and actual participants were also encouraged to share (via snowball effect) the email announcement, with colleagues. Eligibility included anyone aged 18 years and older, able to speak English, and currently practicing as an RN in the United States. Participants were offered a $25 Amazon gift card after participating in the qualitative interview.

A total of 22 RNs agreed to be contacted by the principal investigator (PI) to schedule an interview and were assigned participant numbers. Four (*n* = 4) participants were not interviewed due to inability to follow‐up (*n* = 1), suspicion of imposter participant (*n* = 2), and departing the interview at the time of verbal consent (*n* = 1).

### 2.3 Data Collection

Semi‐structured interviews were conducted via Zoom from January 2024 to February 2024 when data saturation was achieved. All interviews were conducted by the PI, a PhD‐prepared Assistant Professor. The length of the interviews ranged from 13 to 49 minutes. Two of the participants had prior professional relationships with the PI. At the time of the interview, participants could choose to participate in a face‐to‐face interview with the video capture on or with the video capture off. Demographic data collection included age, gender, years practicing as an RN, specialty/area of practice, and highest level of education. Participants were asked three structured interview questions, and follow‐up questions were customized and adapted to accommodate the research topic (Creswell and Poth 2018). The three questions were as follows:

1. Why do you stay in the nursing profession?
2. What do you want from your job or career?
3. This time period is becoming known as the “Great Resignation.” The work and income‐generating landscape has a wide range of opportunities, including remote and hybrid work, gig work, and content creation on social media platforms. How does the nursing profession fit into this?

### 2.4 Ethical Considerations

Institutional Review Board approval was received from the Binghamton University Institutional Review Board (IRB# 00004658). Verbal consent was collected prior to the start of the interview.

### 2.5 Data Analysis and Synthesis

Interviews were recorded via Zoom and an audio recorder. Recordings were then transcribed verbatim manually in the ATLAS.ti version 9 software (ATLAS.ti Scientific Software Development GmbH, Berlin, Germany). Data analysis was performed by the PI and followed a transcendental–phenomenological reduction approach (Moustakas 2009). To ensure data validity, transcript summaries were sent to the participants, and they were asked to review and report if the summary captured the essence of what they shared and/or if they had any corrections (Creswell and Poth 2018). Ten participants responded, and two participants provided additional comments that were added to the transcript and included in the analysis. Credibility was ensured by the researcher reviewing existing literature for evidence that challenged emerging themes (Merriam 2009). The researcher also practiced epoché during the data analysis process through recognition of any judgments and recording them as notes to maintain awareness of them (Moustakas 2009).

### 2.6 Findings

A total of 18 RNs completed the interviews and were included in the data analysis. Participants were primarily female (*n* = 14, 78%). Among all participants, 33% self‐reported bachelor's degrees in nursing (*n = 6*), 44% self‐reported master's degrees in nursing (*n* = 8), and 22% self‐reported a doctorate degree in nursing (*n* = 4). The participant's average age was 43 years, with an average of 17 years of practicing as an RN (see Table 1 for additional demographic characteristics).

**TABLE 1:** Participant practice specialties.

Participant specialties
Accident and emergency unit
Mental health
Pediatric critical care
Pregnant women and children
Med/surg resource pool
Pediatric emergency
Critical care
Family practice
Trauma
Education
Psychiatric and corrections
Surgical nurse
Emergency
Geriatric
Education/community
Case management and utilization review
Research infectious diseases
Family nurse practitioner

Analysis of the data collected revealed five major themes: why I am a nurse, work environment, human needs, autonomy, and COVID. Within each theme, there were several subthemes (see Table 2).

**TABLE 2:** Themes and subthemes.

Themes	Subthemes	Categories
Why I am a nurse	Make a difference Flexibility Pay/Stability	Career change Enjoy day to day Flexibility Pay/stability Why I stay in nursing Passion Help people/Help peers Attached to patients Make a difference/positive impact Make change Motivations Variety
Work environment	Demands of being a nurse Leadership support Workplace violence and incivility	Burnout Communication Healthcare system issues Hospital nursing Leadership support Lunch breaks Mean patients/mean families Mandation Not bedside Patient ratios Poor treatment Retention efforts Staffing Value Unable to give care Workplace violence
Human needs	Respect Value Wellbeing	Appreciated Perceptions of nursing Punitive Respect Professional skills Resources for nurses Pay Wellness/self‐care Work/life balance
Autonomy	Clinical practice Institutional level Schedule	Autonomy Opportunity for growth Become a nurse practitioner
COVID	Staffing Workload	COVID

### 2.7 Why I Am a Nurse

The first theme is why I am a nurse. This theme includes the following subthemes: make a difference, flexibility, and pay/stability. Nurses in this study reported that they always wanted to be a nurse, felt a sense of calling, and were inspired by family members who were nurses. A common sentiment expressed by participants was that they always felt a passion for caring for people.

Participants reported that variety and flexibility in nursing are positive attributes. Within the profession of nursing, someone can work in a variety of fields, settings, and roles. Nursing continues to evolve with roles such as in nursing informatics, telehealth, or in online positions.

…that's what I tell different people when they're kind of contemplating, going into nursing is like you know you don't have to be just at the bedside. You go in thinking that's what you wanna do and your path can lead you into so many, so many different directions. (P19)

Some participants find the profession to be flexible in terms of scheduling:

I like the flexibility of it, um like, I can just work nights and weekends or I can just work a few days every week. I can work Monday through Friday, at like a family practice office, you know there's a lot of flexibility in what you can do. (P6)

Many participants report that as nurses, they make a difference. Nurses feel fulfilled in helping people individually as well as making a difference in the system such as in quality improvement, decreasing workplace violence, workplace bullying, and improving staffing.

I have always felt that I had a job that made a difference. You know whether it was when I was at the bedside and being able to you know help somebody have the experience they wanted in the hospital or maybe save a life…even in academia when I'm not at the bedside anymore I impact people's ability to become a nurse or people's ability to stay in school…I just always had a job that made a difference. (P17)

Pay was reported as both a reason that contributed to why nurses stay in the nursing profession and something that could be improved upon. Many participants reported that the pay was a reason they became a nurse and that they earn a good wage. Others feel that when they examine the importance of their work, education, and level of responsibility, the pay is low compared to other fields. The profession is also stable, and there are always jobs available because of the constant nursing shortage. This shortage has also created opportunities to make more money as an agency/travel RN.

### 2.8 Work Environment

The next theme to emerge was the work environment. Subthemes included the demands of being a nurse, leadership support, and workplace violence and incivility.

Demands inherent to the nursing profession include settings that require staffing 24 hours a day. Nurses have to be willing to work nights, weekends, and holidays. Additional demands on nursing often stemmed from patient load, increasing responsibilities and tasks, liability, lack of meal breaks, and insufficient time off. Participants report having fewer people to do the same amount of work and express frustration with spending two to three hours per shift charting.

You get handed six patients, and yes every single one of them needs everything done and no their dressing didn't get changed during dayshift and now yes you have to change it because it hasn't been changed so, good luck. We're gonna have to wake them up at 2 in the morning because now we actually have time to change the dressing. You know? It sucks, but let's go do it. (P6)

I'm sick of people normalizing not taking a damn break. Excuse my language for anyone that's gonna listen to this. I'm just so passionate about that. Oh, I've worked 12 hours and didn't get a break. That's not cool. That's not something to brag about. That's abuse, like you deserve your lunch. (P16)

…if you look at gen Z and and just the younger generations are doing, they can make a living in shorter periods of time, whereas you've got 12, 13 hour shifts for nurses. And it seems like the research is supporting that that's too long. And the medical errors are made in that period of time because nurses become fatigued and then all at the same time, even though these are mandated, nurses work every single day with the risk of losing their license because those organizations are not gonna stand behind them when a mistake is made because they're doing their fourth 12‐hour shift in a row. (P20)

I think the most frustrating thing for nurses is to not be able to give the care that they feel their patients deserve. That's the worst part of it. They want to care for them. They want to help them. But they are not, they are stuck in the system where they're not able to…I was sad to walk away from it because I absolutely loved working on those patients but…when you don't feel right about what you're doing, you don't wanna do it anymore. (P5)

I can't listen to the patient and have that time to ensure the patent gets effective communication because it will put me behind…they don't want us to take shortcuts as nurses but sometimes it's the choice you're left with. You don't want to, if you really went down that road you really wouldn't be able to finish your work in 12 hours. (P12)

Workplace violence and incivility was identified as a common frustration among participants. Participants reported experiences with incivility with patients/families and unkind colleagues. They discussed how these factors contribute to wanting to leave an already difficult profession. One nurse reported the experience of her adult child who is in the nursing profession:

… my son was punched in the face this week from a patient and you know there's all kinds of issues people deciding your schedule instead of you deciding your own schedule, workplace violence, bullying, and uh you know my daughter‐in‐law has experienced that in the ICU. So that's just I think people want to get away from the bedside. (P20)

We have a ton more injuries. We have had more staff assaults. We have people punched in the face; their nose broken. Like something really bad is gonna happen. (P5)

Lack of leadership support was identified as a factor leading to dissatisfaction. Nurses in the study reported a lack of communication, lack of transparency, conflict with leaders, and leaders not listening to concerns. Participants also expressed challenges with frequent leadership turnover.

The hospitals are gonna have to realize where the problems lie. And the only way they are gonna realize that is by speaking to nurses. And let them tell you why it is that I'm leaving this joint. And I don't mean to say that unprofessionally but if we are able to tell you the reasons why we don't wanna come there or the reasons why we are trying to get out of here maybe they can fix it. Or at least try, start, you know making the steps. Most of the time it's burnout. It's a fact that we're getting too many patients or there's too much responsibility or whatever the case may be. And if they start working on those things it might be, you know, something that somebody might wanna look into. Not me but [laughs]. I am done with the hospital. I am done with that. (P21)

P18 reflects on their love of nursing as they are transitioning from being a nurse employee to starting their own business, “…you know I'm not giving up nursing. I'm not resigning from nursing. I'm resigning from corporate nursing. Institutional nursing, that's the better word. Resigning from institutional nursing.”

### 2.9 Human Needs

Nurses in this study discussed what they need to thrive in their professional work including respect, feeling valued, and well‐being. Participants felt that they spent most of their days inside a hospital, working long, hard, hours, and in general did not feel that they were respected by leadership, professional colleagues, and sometimes patients.

…you are telling them to take their prescriptions or take their drugs… but then the moment the doctor says the exact thing you said like ok this is how you take your drugs and it's like ok they follow it strictly and it's like they, they think that if they don't obey what the doctor said that something bad is going to happen because it is coming from a doctor…the way families look at it is we are just supposed to run around and then carry out on the doctor's instructions and all of that. (P8)

Many participants did not feel that they were valued individually or as a profession. One nurse leader reported that when nurses in their organization are promoted to a leadership position they are often told after they accept the role that there is no office for them.

… you know just that little thing on an institutional level, people don't realize that it makes people feel unvalued when you are, they're like I don't know where I'm going to put you, I don't have a place for you. …I think there are good people in the institution but I think sometimes they don't pause to think how their approach to the situation looks. (P1)

Well‐being and work–life balance were reported as another need. Participants discussed having a high workload, being frequently asked or required to work overtime, and working these extra hours often in short‐staffed conditions. Participants shared ideas to make nursing more expansive through options increasingly offered in other fields such as remote work, flexible work, or telehealth options.

### 2.10 Autonomy

Autonomy in clinical practice, at the institutional level, and over one's schedule was reported as a driver of satisfaction. Some participants reported a lack of autonomy in decision‐making for their patients. This has led some participants to pursue an advanced degree in nursing.

When I moved up into the nurse practitioner with more autonomy and more abilities, capabilities to help individuals, …the ability to do the things that I can do with a little autonomy and a little more respect if you will. I haven't thought about doing anything else. But before that I did….it was before the pandemic, um, and I was probably stuck on 12 hour shifts and having too many patients in the hospital, you know. Having you know you show up to work and somebody didn't show up so now you have ten patients versus 5 but you still have the same responsibilities and all of those things that go along with working in the hospital. Uh, yeah, I would have told you that this is not it. Now I have so, so, decided that I will never work in the hospital again. (P21)

Participants reported that they would prefer to have more autonomy at the institutional level. Some reported that they want the ability to make change, improve nursing and patient care but as a clinical nurse, there is limited ability to do that. This leads to nurses leaving the clinical role to pursue leadership roles. Nurses in this study discussed an increase in satisfaction related to autonomy over their schedules:

People really valued being able to you know if my kid is gonna be in the school play on Thursday and I wanna be there, then I could schedule around Thursday so that autonomy existed way back then. And I think that's why that unit had so many people stay for years. (P17)

### 2.11 COVID

COVID‐19 continued to be an influence in several participant's practices. Some participants recalled disappointment in the level of appreciation they felt when they were working during the early COVID stage. Participants discussed how staffing has not recovered in the years since the pandemic for different reasons such as nurses leaving direct patient care or the profession, and others reported that their institutions have struggled financially since COVID and have cut nursing staff. Workload has also shifted for some participants as they report that “during COVID” more responsibility was given to the RN to reduce staff at the bedside, and once the need to do that passed, the tasks remained with the RN.

### 2.12 Essence of the Phenomenon

In a phenomenological study, after significant statements are clustered into themes, structural and textural descriptions are developed and then integrated into a composite description called the essence of the phenomenon (Creswell and Poth 2018). In this study, RNs practicing during the “Great Resignation” felt disconnected from the passion that inspired them to become a nurse. Participants were dedicated to excellent patient care, but many feel that they were no longer able to do so due to workload, staffing, and inadequate support from their leadership. Nurses value the variability of a nursing career but also feel that the demands are too high to succeed. Participants wanted to be able to make changes and make nursing better for themselves and future generations. The world and the work environment are evolving and nursing is struggling to provide the level of autonomy and flexibility that participants want and need. The effects of COVID have amplified many of these factors and continue to impact the profession.

## 3 Discussion

This study examined the experiences of nurses practicing during and after the time period known as the “Great Resignation.” The findings in this study were analyzed through the lens of the Motivation to Work theory (Herzberg et al. 1993).

During and after the COVID‐19 pandemic, workers from all fields were resigning in record numbers due to stagnant wages, childcare issues, and the workplace environment (Miller and Jhamb 2022). The workplace landscape experienced a significant shift during this time with participants observing growing numbers of workers earning a living from home, as an employee, or starting their own business. Some workers could choose their hours to work around their personal and family lives. Participants reported that the nursing profession has not evolved with that shift, thus a reexamining how they spend their own working hours. This is then intensified by increasing demands, workplace violence, lack of appreciation, and feeling like they no longer have the ability to deliver the care they want due to time constraints related to workload. The very thing that called them to the profession—making a difference in the lives of patients—is lost in the unmanageable day‐to‐day tasks and short staffing (Lessi et al. 2024).

Participants became nurses because they wanted to help people and make a difference. They were committed to their patients and desired to provide high quality care. They stayed in nursing for those reasons and the enjoyment of the day‐to‐day work, which are both linked with the intention to stay (Lee and Lee 2022). Increased workload and staffing interfered with participants’ ability to provide the care that they felt their patients deserved. That, in combination with feeling under‐supported, underappreciated, and undervalued, has made several participants consider or actually leave their workplace. Key factors to retaining experienced nurses are leadership and a healthy working environment, both as independent factors and as interconnected ones (Al‐Hamdan et al. 2017; Loft and Jensen 2020; Raso et al. 2022).

Nurses who are burned out but chose not to leave or are unable to leave may also engage in “quiet quitting”: reducing their efforts to mainly performing tasks that are minimally necessary (Galanis et al. 2023). Quiet quitting has been linked to an intention to leave (Gün et al. 2025). This is significant because nurses who are not quiet quitting but are motivated instead provide higher quality care and improved job satisfaction. Considering this step between burnout and resignation is critical to retaining a high‐quality nursing workforce.

Safety, pay, support, and the ability to care for their patients are baseline needs (Friese et al. 2024). Nurses also need a career trajectory that includes autonomy, flexibility, and opportunities for growth, advancement, and promotion (Jarden et al. 2023; Yasin et al. 2020). Moreover, nurses need to feel valued and treated like an autonomous member of a self‐governing profession in their organization (Bäckström et al. 2024).

Findings from this study were examined through the lens of the Motivation to Work theory (Herzberg et al. 1993). The findings support the theoretical framework stressing that while factors such as salary and baseline working conditions are essential to decreasing dissatisfaction, essential factors in motivating workers to stay are increasing satisfaction through factors including growth and advancement potential, achievement, recognition, and responsibility.

### 3.1 Implications for Nursing and Health Policy

Strategies to retain nurses must allow nurses to feel connected with the reasons that called them into the profession. Findings from this study suggest that policy should focus on ensuring nurses have the resources to deliver high quality care, feel valued, have autonomy, and have opportunities for growth. Staffing needs to be adequate for each of those areas to be addressed. Therefore, retention must be a priority. Retaining nurses will allow leaders to focus on these key areas. Both must be simultaneously prioritized as they drive each other. Staffing should be abundant enough that leaders can provide clinical RNs with opportunities for growth including activities such as chairing committees or participating in research. Time dedicated toward these activities may lead to increased satisfaction and decreased burnout.

### 3.2 Limitations

This study has three limitations. First, while the sample size is adequate for qualitative research, a larger sample size may have revealed additional information or contributed to the thematic analysis; thus, limiting the generalizability of the findings. Second, inherent to qualitative research, and specifically phenomenology, is the potential for researcher bias as the researcher is considered the instrument. This study was conducted by a single researcher which limited the validation of themes. Lastly, participant response to data validity is noted; only 10 of the 18 participants responded to the data validity request.

## 4 Conclusion

The “Great Resignation” is a relatively new phenomenon and the experiences of nurses who remained working during this time period are emerging. Much of the literature available are opinions and editorials with an urgent message of addressing this phenomenon, citing reasons that they believed may be the cause. This study aimed to add to the body of knowledge specific to nurses’ experience during and after this time. Nurses in this study chose nursing because of the drive and passion to help people and make a difference. They feel there are a growing number of barriers to delivering high quality care due to staffing and increased workload. They also feel a lack of leadership support, autonomy, appreciation, and value, all while working long shifts and holidays often with no break for a meal. This occurs while participants are living in the context among workers in other fields enjoying increased flexibility and autonomy while working fewer hours. This study can generate and/or add to the conversations of leaders at all levels to develop strategies and implement policies to improve retention and examine the sustainability and elevation of the nursing profession.

## Author Contributions

*Study design*: Jolene Kittle. *Data collection*: Jolene Kittle. *Data analysis*: Jolene Kittle. *Manuscript writing*: Jolene Kittle. *Critical revisions for intellectual content*: Jolene Kittle.

## Conflicts of Interest

The author declares no conflicts of interest.
